# How to Rescue American Football

**DOI:** 10.7759/cureus.592

**Published:** 2016-04-28

**Authors:** George D Lundberg, David Metzner

**Affiliations:** 1 Chief Medical Officer, CollabRx; 2 Editor at Large, Medscape from WebMD; 3 Consulting Professor, Stanford University School of Medicine; 4 Retired Plastic Surgeon

**Keywords:** football, brain damage, chronic traumatic encephalopathy, concussions, punch-drunk, helmets, safety rules, contact sports, tau protein, prevention

## Abstract

Blows to the head damage the brain. American football is a contact/collision sport that produces many injuries, including to the brain. Football has many supporters who cite important redeeming characteristics of the activity. Public attention to the hazards of children and adults playing football has heightend recently due to many new scientific discoveries, not least of which is the frequency with which players are seriously harmed and do not recover. It is now incumbent on all interested parties to invent and implement far better safety practices, equipment, rules, and processes or the sport must cease to exist in its current form. This paper presents several safety proposals for consideration and study.

## Editorial

American football is a great sport. It offers, requires, nurtures, and rewards speed, skill, strength, cunning, offensive and defensive strategic thinking, courage, judgment under pressure, competitive spirit, reliance on teamwork, a requirement for exquisite timing, and resolve. Football teaches a participant how to get up and get back at it after being knocked down again and again, a great life lesson. And football has the capacity to engender huge dedicated fan bases.

Unfortunately, as the supreme contact sport, it is also a collision sport and thrives on a degree of inherent violence. Thus, injuries are expected and common. Most heal, usually without any disability. Some do not.

Studies by US government agencies, the Centers for Disease Control (CDC) and the National Institute for Occupational Safety and Health (NIOSH), have found that those football players exposed to the ultimate of potential violence, professionals in the National Football League (NFL), live longer lives, on average, than men of their birth cohort, but that they have greater likelihood of chronic dementia than does their age cohort. [Brain and Nervous System Disorders Among NFL Players. (2013). Accessed: April 4, 2016. http://www.cdc.gov/niosh/pgms/worknotify/pdfs/NFL_Notification_02.pdf]

Based upon the foregoing and other information, other observers have proposed new rules of play to improve football safety in general. The following seem sensible to us:

Kickoffs should be eliminated, except perhaps at the beginning of each half, and instead, the ball should be placed at the 20-yard line after touchdowns and field goals, and at the 35-yard line after safeties. The most violent collisions occur when the players are traveling at full speed in opposite directions when colliding. This kickoff rule change would sharply diminish the most dangerous and injury-causing collisions while not fundamentally altering the essence of the game.

One study from Boston University published in 2015 found that later life cognitive impairment was found only in football players who played tackle football under the age of 12 years [1]. Based on this study, and common sense, it seems an easy decision to replace all helmeted tackle football for participants under the age of 12 years with flag football.

While injuries of a wide range of types are inevitable, they are generally manageable. Those responsible for safety in the sport seem (finally) to be actively engaged in preventing occurrence and complications of cerebral concussions by stricter enforcement of stronger rules for the prevention, diagnosis, and clinical management.

A study from the Mayo Clinic published in 2015 did show that one of three persons at autopsy who had played contact sports had tau protein (characteristic of  chronic traumatic encephalopathy (CTE)) found in their brains. No tau was found in brains of individuals who had not played contact sports [2]. Greater vigilance and diligence remain essential.

The issue of multiple sub-concussive blows as a cause of chronic traumatic encephalopathy remains enigmatic. It has been proposed that regular positioning of the defensive front seven in stand-up mode at the snap rather than 3 or 4-point stances could sharply reduce the every play, often helmet to helmet, sub-concussive clashes of linemen.

Then, we must return inevitably to the helmet itself. Originally designed many decades ago to prevent skull fractures, headgear has evolved and enlarged to prevent broken noses, fractured cheekbones and orbits, and to protect the eyes, the jaw, and (along with mouthpieces) the teeth. Helmets generally do work well for those purposes.

Some have suggested that we eliminate the helmet and other padding and convert American football into a rugby-like sport. Many observers say that they have not seen head/brain damage in rugby. That may simply be inexperience or selection bias speaking. Indeed, in the best recent meta-analysis of concussions from sports published in 2015, rugby produced eight times the incidence of concussions as American football and four times that of hockey [3]. So, helmets do help in many ways.

But the current rigid helmet fits very snugly to the skull, requiring the helmet and head to move as one. That is a big problem for the movable brain.

In boxing, we know that rapid acceleration and deceleration of the movable brain within the rigid skull, produced by blows from a fist, especially when angular, or the skull hitting the canvas floor, induces concussions and often serious structural and functional brain damage [4]. 

This also probably produces chronic traumatic encephalopathy resulting from multiple sub-concussive blows.

### Proposal for new football helmet design

To protect from those forces, we propose a radical new design for football helmets.

Football helmets currently in use consist of a solid unyielding shell inside of which the skull is firmly constrained so that the helmet and skull move and stop moving, namely, accelerate and decelerate, in unison. The brain, meanwhile, is like a loosely tethered raft in a turbulent and disorganized sea, rolling, pitching, and banging against the rocks. The consequence is that while football helmets succeed in protecting against skull fracture, they do not reduce and may contribute to the risk of traumatic brain injury.

We believe it is necessary to eliminate the rigid outer layer that causes the helmet and skull to decelerate and/or accelerate instantly when impacting the ground, a knee, or another helmet, often followed instantly with a rebound in the opposite or angular direction. A helmet that is compressible will spread the impact over a longer period of time and thereby reduce both the force and kinetic energy of the blow. In other words, the impact occurs in relatively slow motion. That quality of dampening the blow and reducing helmet/skull deceleration and acceleration will ameliorate brain momentum and inertia. Using the metaphor of the brain as a raft, the sea in which it floats would be calmer. The simplest way to think about this football helmet design is that it becomes a shock absorber.

We propose, as an alternative, silicone gel within a flexible silicone shell. Silicone gel has the property of absorbing impact, does not transmit vibration, and is unaffected by ambient temperature. Throw a baseball and a breast implant against a brick wall and observe what happens. The ball will rebound with barely diminished force and speed. The implant will merely fall to the floor. As opposed to the highly viscous silicone in pressure-reducing pads that resist flowing, the flow of less viscous silicone gel is an essential property of this helmet design. Experimentation would determine the best gel viscosity and shell thickness. Depending on the shell characteristics, an additional outer and inner cover can be added to prevent tearing. There can be an external surface layer of fabric-like material, perhaps incorporating Teflon, the purpose of which is to reduce friction and to display team colors and logos. An inner layer of solid material would provide structure and attachment points for face masks and chin straps. Durability, that is, the ability to take blow after blow without loss of its physical qualities, would be very high. The outer fabric could be easily replaced, as could the silicone layers. If other energy absorbing materials are proved effective, they could be also be layered inside. The final design would then be one where the overall benefit exceeds the sum of the parts.

Granted, this helmet design is conceptual. However, it is based on scientific fundamental principles of physics (details available on request) and on the experience of one of the authors (Metzner) with silicone gel. Research and development will determine its validity, the ideal gel viscosity, and thickness. We believe that the volume required to provide protection will not result in overall mass compared to current helmets. Rather, we believe that it can be produced in a way to closely resemble the appearance of the current helmets. In other words, the construction and performance would be radically different, but not the appearance. 

With a mix of full disclosure of scientifically determined risks to all involved, rule changes, such as those proposed or detailed here, more stringent enforcement of safety rules on the field (and in the front offices), clear and truly informed consent from participating adults or parents, and a new and improved helmet design and engineering, we may be able to sustain this great sport for a long time to come.
